# Pan Shuh: the founding figure of modern Chinese psychology

**DOI:** 10.1093/procel/pwad064

**Published:** 2024-01-17

**Authors:** Ligang Wang, Tongqi Wei, Liang Wang

**Affiliations:** Institute of Psychology, Chinese Academy of Sciences, Beijing 100101, China; CAS Key Laboratory of Mental Health, Institute of Psychology, Beijing 100101, China; Institute of Psychology, Chinese Academy of Sciences, Beijing 100101, China; Institute of Psychology, Chinese Academy of Sciences, Beijing 100101, China; CAS Key Laboratory of Mental Health, Institute of Psychology, Beijing 100101, China; Department of Psychology, University of Chinese Academy of Sciences, Beijing 101408, China

Entering the lobby of the Institute of Psychology, Chinese Academy of Sciences, the first thing that catches the eye is the Pan Shuh Library. In the library, Pan Shuh’s six decades of life are displayed in the form of a life photo exhibition, representing 10 years of determination, 10 years of wandering, 10 years of exploration, 10 years of following the path, 10 years of self-improvement, and 10 years of propagation. These six decades not only reflect the life journey of Pan Shuh, the pioneer of Chinese psychology but also serve as a microcosm of the historical development of modern Chinese psychology (Qicheng Jing, Xiaolan Fu, 2011). Pan Shuh was one of the founders of modern Chinese psychology, a trailblazer in theoretical psychology, and one of the creators of the Institute of Psychology of the Chinese Academy of Sciences and the Chinese Psychological Society. He was also a major founder and leader of the “Jiusan Society” and the China Association for Science and Technology. He once served as the Vice Chairman of the Central Committee of Jiusan Society, the Chairman of the Chinese Psychological Society. In 1955, he was elected as a member of the Academic Divisions of the Chinese Academy of Sciences.

**Figure 1.**
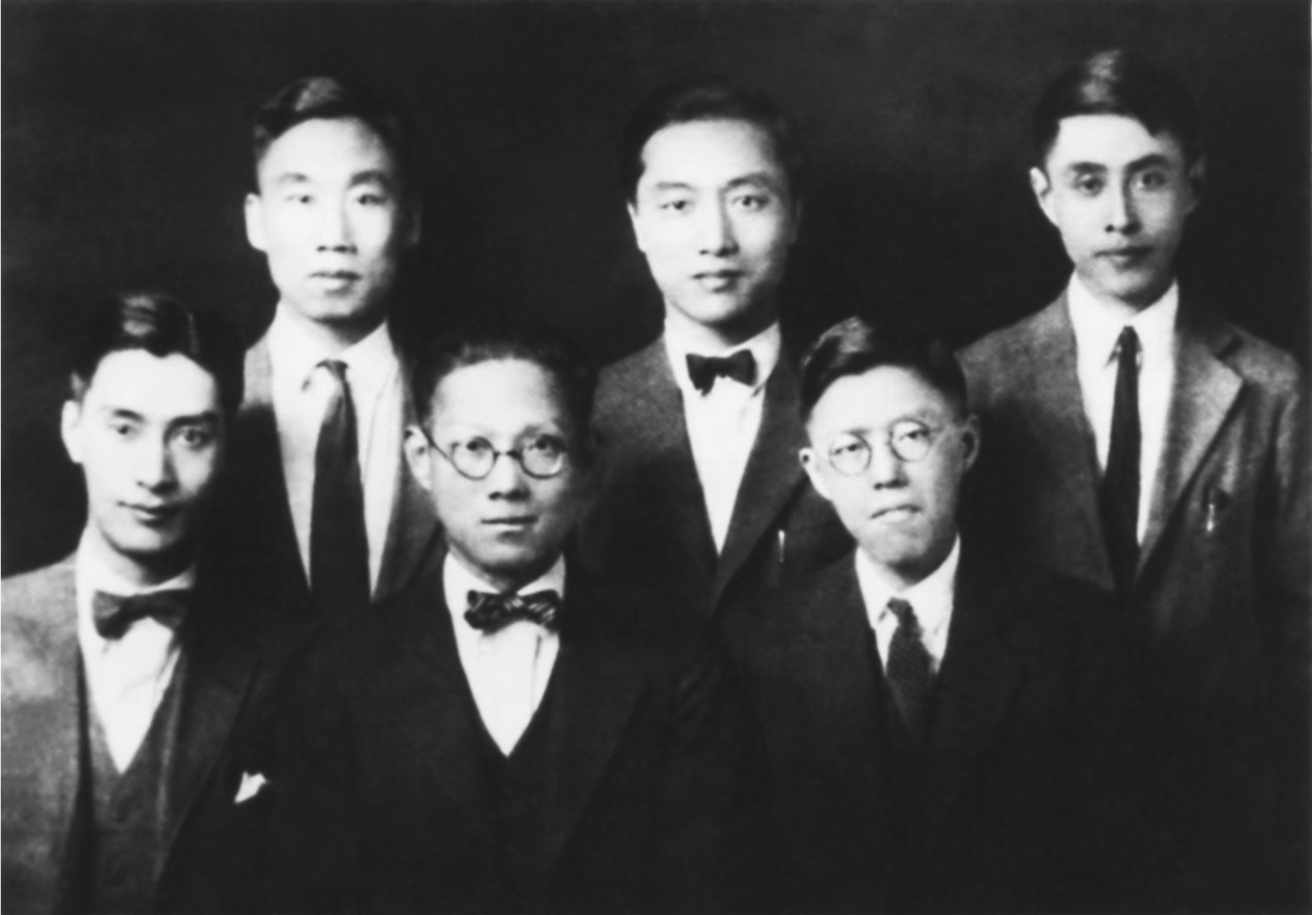
**Pan Shuh (The 2rd from left, line 2) studied at the University of Chicago.** The figure was provided by Pan Shuh’s son Pan Ningbao and his daughter-in-law Chen Shaoying.

Pan Shuh （潘菽）, whose given name is “Younian” with the courtesy name “Shuishu,” was born in 1897 in a scholarly family in the village of Luping, Yixing City, Jiangsu Province, China. He was diligent and eager to learn since childhood, and in 1917, he was admitted to the Philosophy Department of Peking University. After graduation, he was qualified for the government sponsorship of Jiangsu Province and began his 6-year study abroad in the spring of 1921. During his study at Peking University, Pan Shuh was deeply inspired and influenced by the lectures of American educator Dewey, and formed the ideal of saving the country through education. Initially, Pan Shuh chose education during his study in the United States. However, after a period of time, he felt that American education might not be able to solve China’s problems. He then met fellow Chinese students like Cai Qiao and Guo Renyuan, who were senior students majoring in psychology. Through discussions and exchanges with these seniors, Pan Shuh developed a fondness for psychology, believing that psychology, as the science of studying human beings, had extraordinary significance. He thought that psychology possessed a more fundamental nature than education. Thus, he changed his major to psychology, embarking on his journey in this field. Under the guidance of Harvey A. Carr, Pan Shuh obtained his doctoral degree from the University of Chicago in 1926. He retrospectively described this phase as a decade of determination (Fig. 1).

**Figure 2.**
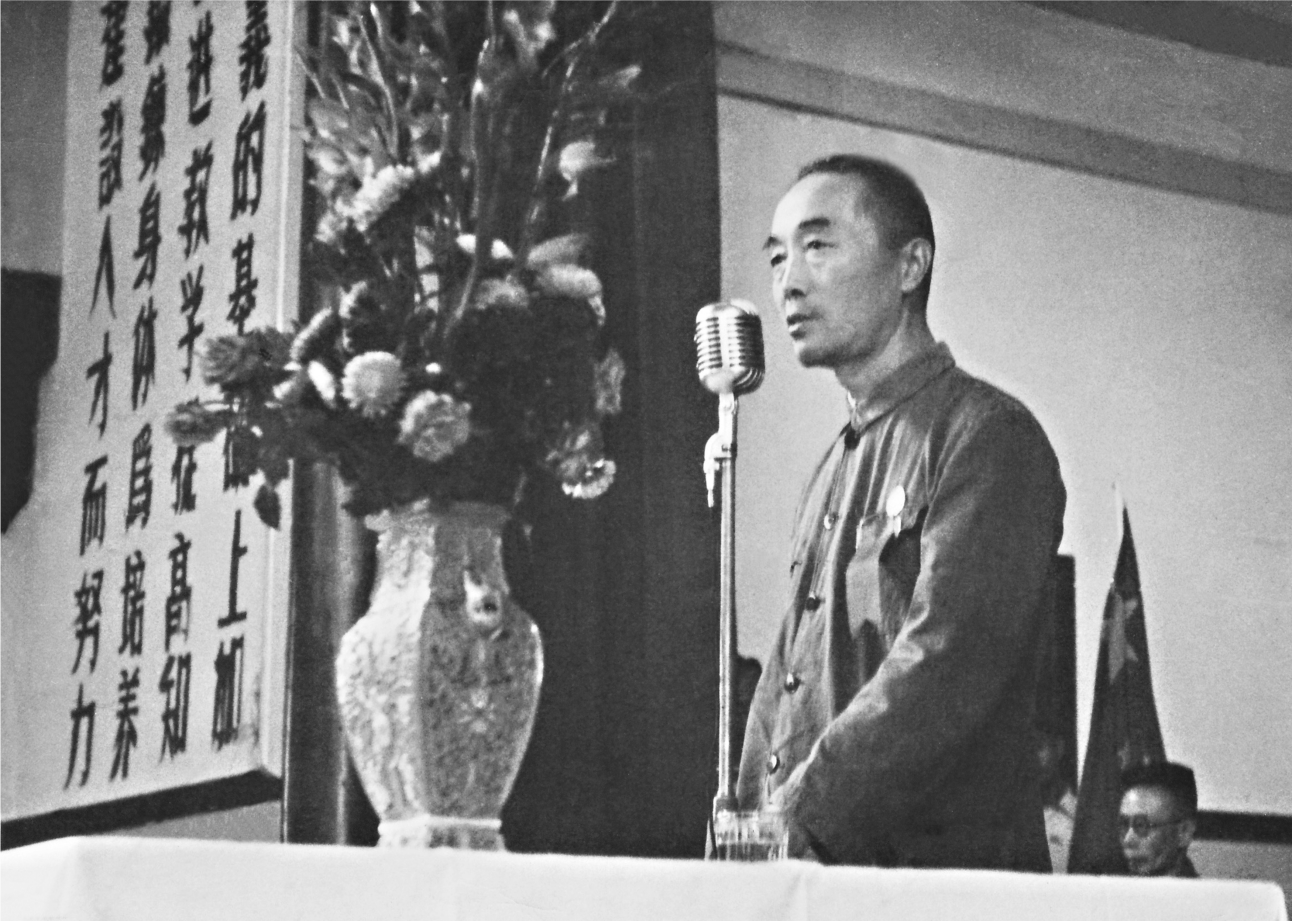
Pan Shuh was giving a report when he was the president of Nanjing University.

In 1927, after returning to China, Pan Shuh was immediately appointed as an associate professor at the newly established National Fourth Sun Yat-sen University, and within half a year, he was promoted to a professor and became the head of the Psychology Department. In 1928, the university was renamed National Central University. Before the liberation, Pan Shuh remained at the College of Science, where he taught over ten courses, including General Psychology, Experimental Psychology, Theoretical Psychology, Physiological Psychology, Comparative Psychology, and Applied Psychology. He never relied on ready-made textbooks but instead created his own lecture materials, characterized by independent insights and a well-structured system. Due to his pedagogical background, he believed in guiding students rather than spoon-feeding them, encouraging independent thinking and activating their enthusiasm and initiative. In the eyes of his students, Pan Shuh was not only a great teacher but also a helpful friend. Professor Huang Naisong of Suzhou University, who was once a student of Pan Shuh, recalled, “I cannot describe how he taught me, but I always felt that he was constantly educating me. A complimenting nod, a disapproving expression, all provided guidance on how to distinguish right from wrong and how to be a better person.” After the liberation of Nanjing, National Central University was renamed Nanjing University, and in November 1949, Pan Shuh became the chairman of the university’s administrative committee. When the university changed to a presidential system in 1951, Pan Shuh was appointed as the first president of Nanjing University (Fig. 2). During this period, China faced serious difficulties in economic and scientific development. Psychology also suffered greater setbacks. Many universities abolished psychology departments, and numerous psychologists changed their career paths, putting the young discipline of psychology in China at risk. However, in the face of the potential demise of psychology, Pan Shuh’s confidence in the field remained unwavering. Following the suggestion of his brother Pan Zinian, Pan Shuh seriously studied Marxist-Leninist theory and gradually formed the branch of theoretical psychology, exploring a new path for the development of psychology in China. From 1927 to 1949, Pan Shuh entered a decade of wandering and exploration.

**Figure 3.**
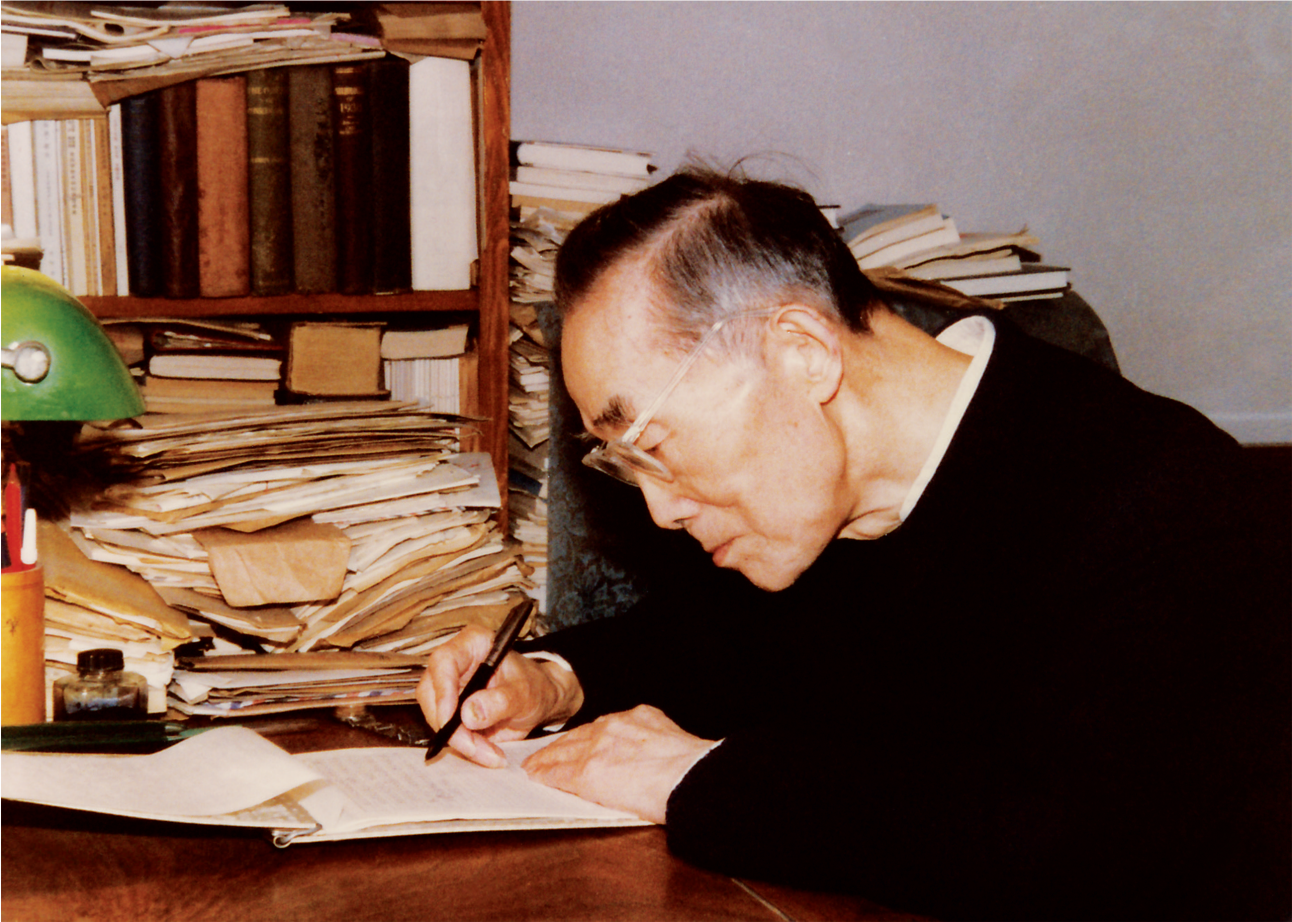
**Pan Shuh was still writing at the age of ninety and working tirelessly at his desk.** The figure was provided by Pan Shuh’s son Pan Ningbao and his daughter-in-law Chen Shaoying.

During the period from 1955 to 1956, according to the planning of the national education department, the Psychology Department of Nanjing University merged with the Psychology Research Office of the Chinese Academy of Sciences, expanding into the Institute of Psychology. As the president of Nanjing University, Pan Shuh focused on the overall development of psychology in China and chose to integrate with the Psychology Department. In 1956, Pan Shuh, together with the entire faculty and all resources, relocated the Nanjing University Psychology Department to Beijing, establishing the Institute of Psychology of the Chinese Academy of Sciences, with Pan Shuh serving as its director. Pan Shuh’s lifelong academic pursuit was to enhance the scientificity of psychology while he still insisted that psychology possessed both the characteristics of natural science and social science. This dual nature was an essential feature of psychology, and the two aspects could not be separated or isolated from each other. Under the cover of writing “inspection” materials, he secretly wrote a preliminary draft of “Psychological Digest,” totaling 500,000 words. Those who had the opportunity to read the manuscript were deeply moved by Pan Shuh’s spirit of tirelessly striving for the survival and future development of psychology in adversity (Fig. 3). From 1955 to 1976, Pan Shuh entered a decade of perseverance and self-improvement.

In June 1977, the Institute of Psychology of the Chinese Academy of Sciences was formally restored, and Pan Shuh resumed his position as director. In his late eighties, Pan Shuh bravely and resolutely shouldered the heavy burden of rebuilding Chinese psychology. In August 1977, the National Psychology Discipline Planning Symposium was held in Pinggu, Beijing, and Pan Shuh personally presided over and formulated the preliminary plan for the discipline. This plan was arranged in three stages: a 3-year plan, an 8-year plan, and a 23-year plan (1977–2000), which greatly promoted the recovery and development of psychology in China, inspiring psychologists across the country. This plan was regarded as a significant turning point and milestone in the development of psychology in China. Despite his advanced age, Pan Shuh authored over 60 academic papers, reports, and speeches in the last decade of his life, and he published several works, including “Educational Psychology,” “Human Intelligence,” “Selected Works of Pan Shuh in Psychology,” and “Study of Ancient Chinese Psychological Thought.” Even on his deathbed, Pan Shuh was preoccupied with unfinished work. Unable to speak, he wrote “Open the drawer.” Inside the drawer, there was his reply to Tang Zijie from Chongqing Normal University and an article evaluating Maslow. His dedication to the cause of psychology for the party, the people, and his beloved field persisted until his final moments (Chinese Psychological Society, Institute of Psychology of Chinese Academy of Sciences., 2007).

**Figure 4.**
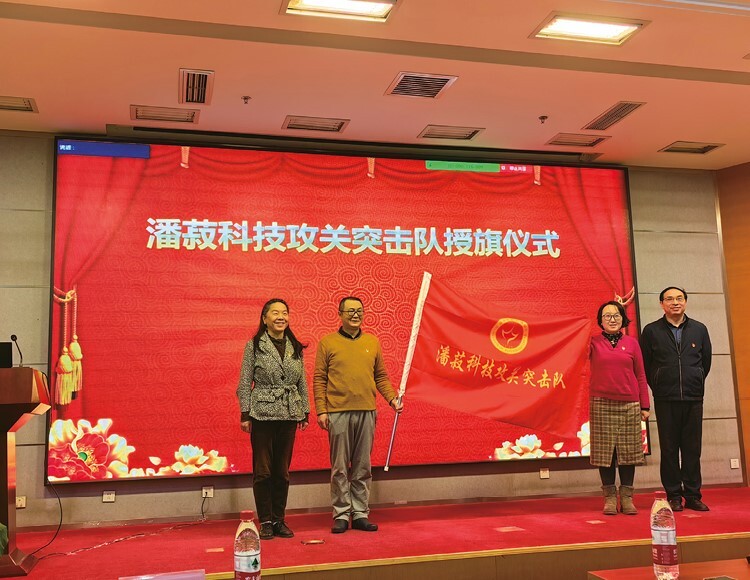
Flag Awarding Ceremony for Panshuh Team of Science, Technology and Innovation.

Pan Shuh’s psychological ideas had already formed his own theoretical system, which had a profound and extensive impact not only in China but also in the international theoretical psychology community. Statues of Pan Shuh stand tall at Nanjing University and his hometown, Yixing City, Jiangsu Province. He is praised with various honorable titles, such as the “Master of Chinese Psychology,” “A Symbolic Figure of Chinese Psychology,” and “An Important Founding Figure of Chinese Psychology.” In order to carry forward and inherit Pan Shuh’s spirit as a scientist, the Institute of Psychology of the Chinese Academy of Sciences established Panshuh Team of Science, Technology and Innovation on 13 January 2023 (Fig. 4). The members of the Attack Team are determined to benchmark the spirit of the scientists of the older generation, always keep in mind the mission of the national team of psychology science and technology, and focus on original scientific research and technological innovation in psychology, and make greater contributions to realizing the country’s self-reliance and self-improvement in science and technology.
